# From IMU Streams to Real-Time Decisions: Past-Only Next-Window Badminton Action Prediction

**DOI:** 10.3390/s26123651

**Published:** 2026-06-08

**Authors:** Qinglin Zhu, Jiao Wang, Bin Guo

**Affiliations:** 1 College of Electrical Engineering, Sichuan University, Chengdu 610065, China; zhuqinglin@stu.scu.edu.cn; 2 College of Mechanical Engineering, Sichuan University, Chengdu 610065, China; wangjiao95@stu.scu.edu.cn

**Keywords:** badminton, action prediction, inertial measurement unit (IMU), wearable sensing, time-series classification, real-time inference, principal component analysis (PCA), BiLSTM, self-attention

## Abstract

We study real-time *next-window* badminton action prediction from wearable IMU streams where the system must predict the action label of the upcoming 100 ms window using *past-only* (causal) information. To handle severe class imbalance in continuous streams, we employ window-level downsampling of the dominant background class and compress multi-sensor time/frequency features using PCA before temporal modeling. We evaluate the full pipeline under a hop-based streaming protocol and show that our BiLSTM + MHSA model achieves high recognition performance (test accuracy 96.36%, Macro-F1 95.82%) while remaining deployable in real time, reaching 58.20 windows/s end to end (including preprocessing), i.e., 5.82× the real-time requirement (10 windows/s under a 100 ms output interval), on a Windows PC with an NVIDIA RTX 3080 GPU. These results support low-latency applications such as live coaching feedback and tactical analytics.

## 1. Introduction

Wearable sensing has become a practical foundation for fine-grained sports analytics, enabling an automated understanding of athletes’ actions, tactics, and biomechanics from lightweight on-body sensors. In badminton, inertial measurement units (IMUs) can capture fast and subtle motion cues of strokes, and recent datasets and studies have demonstrated the feasibility of recognizing rich shot categories from multi-sensor IMU streams. In this paper, we focus on a badminton IMU dataset containing 11 shot types plus an “other” class, recorded at 100 Hz with five IMU placements (lower, upper, left foot, right foot, and racket), yielding 30 raw channels (three-axis accelerometer + three-axis gyroscope per device).

Beyond offline recognition, many real-world applications (e.g., real-time coaching, tactical feedback, and downstream control) require *predictive* inference: the system should anticipate what action is happening in the immediate future rather than only classifying the past. We therefore study *next-window action prediction*: given a history of past windows, the model predicts the action label of an upcoming window using a strict *past-only* (causal) formulation. Concretely, with a 100 Hz IMU stream, the system outputs one prediction every 100 ms, enabling continuous online inference without accessing future observations. This hop-based protocol requires a minimum throughput of 10 windows/s for real-time operation; in our end-to-end stream-replay benchmark (feature extraction, PCA, and model inference), the pipeline reaches 58.20 windows/s (5.82× real time) on a Windows PC equipped with an NVIDIA RTX 3080 GPU. This setting naturally aligns with online deployment, where the current/future window is not fully observed when the decision must be made.

Next-window prediction for badminton IMU is challenging for several reasons. First, badminton motions are highly dynamic with abrupt state transitions; discriminative patterns may be short-lived while still depending on longer-term context. Second, many strokes exhibit similar short-term signatures (e.g., clear vs. smash), making long-range temporal cues important for disambiguation. Third, the data distribution is strongly imbalanced: the background “other” class can dominate the stream, while rare strokes appear sparsely, which can bias training and degrade minority-class performance. Finally, obtaining accurate frame-/window-level labels for such high-frequency sensor data is labor-intensive, motivating learning and labeling strategies that reduce human annotation cost.

To address these challenges, we propose an LSTM-based pipeline for IMU next-window prediction. We first transform each IMU window into a compact representation via multi-channel time/frequency-domain features (13 features per channel) and apply standardization followed by PCA for dimensionality reduction, yielding an *m*-dimensional embedding per window. We then construct a past-only sequence of length *H* and feed it into a temporal model combining BiLSTM-based sequence encoding with multi-head attention and an MLP classifier head. This design targets both short-term variations and long-term dependencies, matching the nature of badminton motion streams. In addition, to alleviate labeling cost, we adopt a self-supervised labeling approach derived from LIMU-BERT-style IMU representation learning, which can generate reliable labels and significantly reduce manual annotation overhead.

The main contribution of this paper is a complete causal prediction pipeline for badminton IMU streams. We formulate the task as strict *past-only* next-window prediction, where the system outputs one prediction every 100 ms without accessing future observations. To support this setting, we combine multi-channel time/frequency features, PCA-based compression, and a lightweight BiLSTM + MHSA temporal encoder that captures both short-term stroke dynamics and longer-range motion context. We further evaluate deployability with a full end-to-end streaming benchmark, including feature extraction, standardization, PCA, and model inference, and show that the pipeline reaches 58.20 windows/s, or 5.82× the real-time requirement, on a Windows PC with an NVIDIA RTX 3080 GPU. Finally, because continuous badminton streams are dominated by the *other* class, we incorporate window-level downsampling and ablation analyses to clarify how imbalance handling, PCA dimensionality, and attention affect prediction robustness, especially at longer horizons.

The remainder of the paper is organized to make this pipeline explicit. Section 3 describes the dataset, preprocessing, temporal model, and labeling strategy, while Section 4 evaluates prediction accuracy, real-time feasibility, calibration, and ablation results. This organization connects each methodological component to the deployment requirements of low-latency badminton analytics.

### Abbreviations

For clarity, the main acronyms used in this paper are summarized as follows: IMU, inertial measurement unit; PCA, principal component analysis; LSTM, long short-term memory; BiLSTM, bidirectional LSTM; MHSA, multi-head self-attention; MLP, multilayer perceptron; ECE, expected calibration error; UWB, ultra-wideband; IoU, intersection over union; GT, ground truth; and PR, precision–recall.

## 2. Related Work

### 2.1. Wearable Sensing for Badminton Analytics

Wearable IMU sensing has been widely adopted for sports motion analysis due to its low cost, portability, and high temporal resolution. For badminton, prior work has demonstrated effective stroke recognition from wearable IMUs (and sometimes additional modalities such as UWB) [1,2,3,4,5,6]. In parallel, forecasting in badminton has been studied at the match/event level, e.g., movement forecasting [7] and rally-wise behavior imitation from offline match trajectories [8]. More broadly, wearable inertial sensing has also been explored for anticipatory/predictive inference beyond badminton, including motion intention prediction [9], forecasting of future gait patterns from IMUs [10], and online frameworks that couple action recognition with motion prediction for early risk assessment [11].

This paper builds on the publicly released badminton wearable-sensing dataset introduced by Van Herbruggen et al. [1]. Their work collected synchronized IMU/UWB recordings from badminton players and demonstrated the feasibility of wearable-based badminton shot recognition under the original dataset protocol. In that protocol, the goal was to recognize badminton actions from the available sensor recordings using the dataset’s original sensing configuration and label definition, providing an important foundation for subsequent badminton IMU studies. Building on this dataset, we reformulate the problem from offline shot recognition to causal next-window prediction. Specifically, our setting differs in sensing configuration, class taxonomy, task definition, and evaluation protocol: we use the five-IMU subset and predict the upcoming IMU window from past windows only under a streaming protocol. Therefore, the recognition accuracy reported by Van Herbruggen et al. [1] is treated as related context rather than as a head-to-head baseline; a controlled numerical comparison would require rerunning the prior recognition model under the same sensor subset, 12-class label set, past-only input constraint, next-window target definition, and train/validation/test split. Compared with these lines of work, our focus is a strictly causal sensor-stream setting that directly supports low-latency online feedback.

### 2.2. Real-Time Constraints and Temporal Modeling for Wearable IMU Streams

In real-time wearable IMU systems, low-latency inference under strict causal constraints is a primary requirement: models must process streaming signals online and output stable predictions within tight timing budgets. Recent reviews of intelligent wearables summarize multiple real-time deployment cases for motion-intent and biomechanical inference [12,13]. Capturing both short-term discriminative cues and long-range context is crucial for highly dynamic motions such as badminton strokes. The Long-Short Term Memory (LSTM) [14,15] framework was proposed to separate fine-grained variations from longer contextual dependencies in time series under complex interventions, providing a principled approach to modeling dynamics. Real-time wearable pipelines have also demonstrated simultaneous action recognition and whole-body motion/dynamics prediction in online settings [16]. Attention-based LSTM sensor-fusion studies further show robust performance under challenging NLOS conditions, reinforcing the practical value of temporal modeling for real-time wearable systems [17]. Recent multimodal wearable forecasting research further reports strong continuous multi-step-ahead biomechanical prediction using a transformer-style encoder–decoder architecture (KsFormer) [18]. Building on this motivation, we adopt an LSTM-inspired design that leverages BiLSTM encoding and multi-head attention to aggregate historical information, and we tailor it to the strict past-only next-window prediction objective.

### 2.3. Self-Supervised Learning and Automatic Labeling for IMU Data

Self-supervised learning (SSL) has emerged as a transformative paradigm for IMU sensing, enabling robust representation learning from massive unlabeled streams and significantly mitigating the reliance on labor-intensive manual annotation [19,20]. The foundational work of LIMU-BERT [21] first demonstrated that masked modeling objectives could effectively unlock the latent temporal patterns in IMU sequences. Building upon this, recent universal models such as oneHAR [22] have further scaled these representations across diverse sensor modalities and datasets. More recently, bio-inspired SSL frameworks [23] have refined this process by incorporating movement-specific inductive biases into the pre-training phase. Inspired by these advancements in automated labeling and feature extraction [24], we propose a self-supervised labeling strategy specifically tailored for badminton dynamics. This approach generates high-fidelity, window-level labels that demonstrate strong empirical alignment with ground truth across various subjects, ensuring both data efficiency and labeling precision.

## 3. Research Methods

This section describes the complete construction of the proposed next-window prediction pipeline. We first specify the dataset source and the IMU-only experimental subset; then, we define how continuous sensor streams are converted into sliding-window samples under a past-only constraint. We next describe the treatment of class imbalance, feature standardization and PCA compression as well as the temporal prediction model used to map historical IMU windows to future action labels. Finally, we introduce the self-supervised labeling component used to reduce annotation effort and support scalable window-level supervision.

### 3.1. Dataset Overview

The dataset used in this study was originally collected and released by Van Herbruggen et al. [1]. We did not collect new player data; instead, we used the IMU-only subset of this badminton dataset obtained from the original authors for reproducible evaluation. The original dataset contains wearable-sensing recordings from three experienced badminton players performing multiple stroke categories with an additional *other* label assigned to periods in which no target stroke is executed.

For the experiments in this paper, we use the annotated IMU-only CSV recordings available for the three players. The recordings are sampled at 100 Hz using Axivity AX6 inertial sensors. Five sensors are placed on the player/equipment system: lower body/waist, upper body, left foot, right foot, and racket. Each sensor provides tri-axial acceleration and tri-axial angular velocity, resulting in 5×6=30 raw IMU channels per time step (denoted as $D_{0}=30$). This multi-position setup captures both whole-body movement and racket-specific motion, which is important for distinguishing fast badminton strokes with similar local motion patterns.

**Figure 1:**
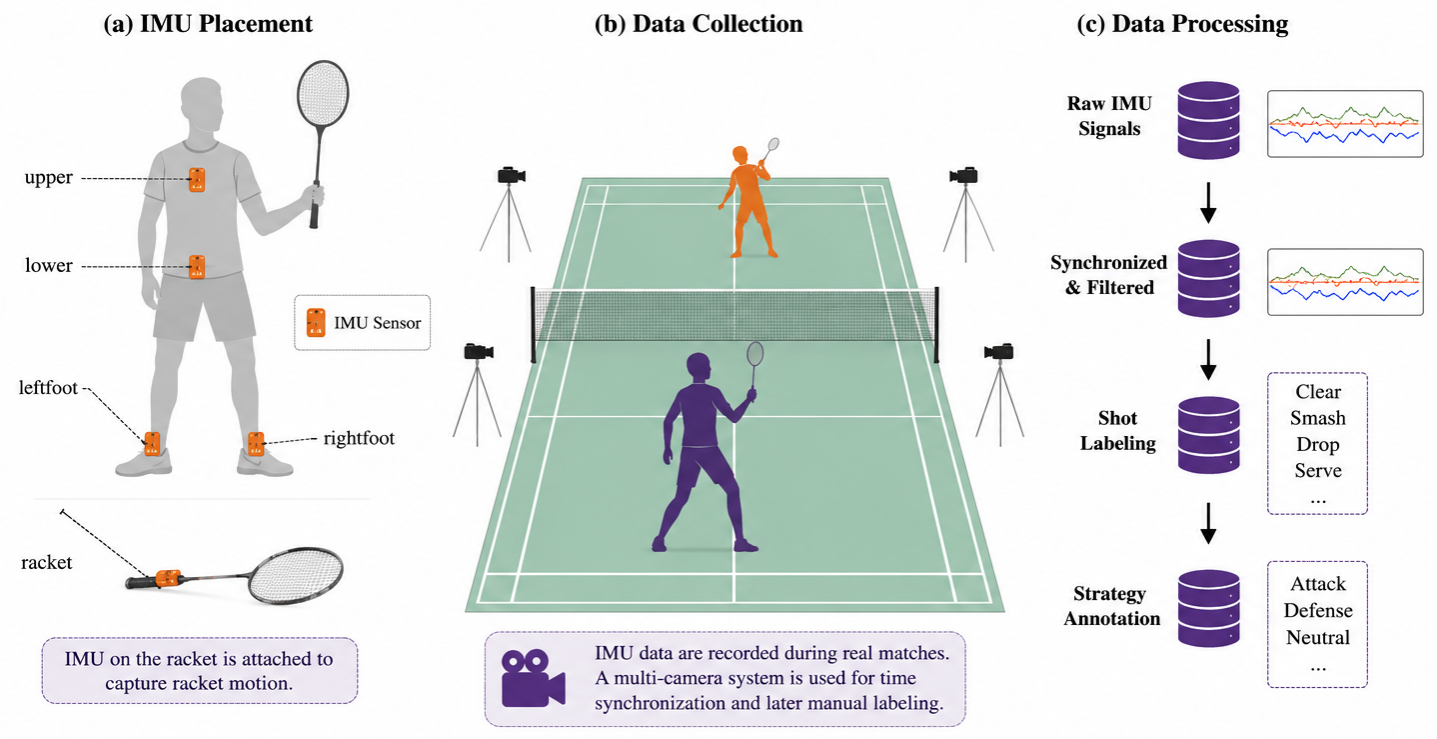
Illustration of the badminton IMU dataset workflow used in the source dataset from Van Herbruggen et al. [1]: (**a**) IMU placement on the upper body, lower body/waist, left foot, right foot, and racket; (**b**) data collection during real badminton matches with multi-camera time synchronization and later manual labeling; and (**c**) data processing from raw IMU signals to synchronized and filtered streams, shot labeling, and strategy annotation. The colored curves in panel (**c**) schematically represent multi-channel IMU signals.

Following the labels available in the released IMU-only annotations, our final experimental setting is formulated as a 12-class next-window prediction task, consisting of 11 badminton action classes and one *other* class. This distinction separates the broader label inventory described in the source dataset from the specific label set used in our reproducible IMU-only experiments. Figure 1 illustrates the data-collection workflow and wearable sensor placement used in the source dataset.

### 3.2. LSTM Model Application

Badminton motions exhibit high dynamics and abrupt state transitions, posing significant challenges for temporal sequence models to extract discriminative features from IMU data. The long short-term memory (LSTM) model is a specialized temporal sequence modeling framework designed to capture both short-term fine-grained variations and long-term contextual dependencies in dynamic time-series data—an advantage that makes it well suited for processing badminton IMU signals characterized by rapid strokes, smashes, and drops.

Inspired by the BiLSTM open-source model proposed by Cai et al. [15], we learn motion representations from the badminton IMU dataset. This subsection introduces the application process and evaluates the ability of the model to capture the dynamic features of badminton motion.

#### 3.2.1. Data Processing

Each recording is treated as a continuous labeled IMU stream

(1) $$\begin{array}{cc} D & ={\left\{\left(s_{t},y_{t}\right)\right\}}_{t=0}^{T-1},\;s_{t}\in R^{D_{0}},\;y_{t}\in Y,\\ \; & \;\phi :Y\to \{0,\ldots ,C-1\},\;C=12. \end{array}$$

The goal is to construct prediction samples that respect the causal nature of online deployment: at prediction time, only historical sensor observations are available, whereas the target label belongs to the upcoming window.

To transform the continuous stream into model inputs, the IMU sequence is divided into overlapping windows of length *W* with hop size hop. The *k*-th window starts at $s_{k}=k\cdot hop$ and is represented as

(2) $$S^{\left(k\right)}=\left[\begin{array}{c} s_{s_{k}}^{⊤}\\ s_{s_{k}+1}^{⊤}\\ \vdots \\ s_{s_{k}+W-1}^{⊤} \end{array}\right]\in R^{W\times D_{0}},\;N_{w}=\left\lfloor \frac{T-W}{hop}\right\rfloor +1.$$

The window label is assigned according to the label at the window end,

(3) $$y_{k}=\phi \left(y_{t_{k}}\right),\;t_{k}=s_{k}+W-1.$$

For next-window prediction, the sample at index *k* is formed from previous windows only, while the supervision target corresponds to a future window. This design prevents information leakage from future observations and matches the intended streaming inference setting.

Each window is converted into a compact multi-channel representation. For channel $c\in \{0,\ldots ,D_{0}-1\}$, let $x^{\left(k,c\right)}\in R^{W}$ denote the one-dimensional signal segment in window *k*. We summarize this segment using 13 time- and frequency-domain descriptors:

(4) $$\begin{array}{cc} \psi (x) & =\left[\mu ,\;\sigma ,\;x_{\min},\;x_{\max},\;\mathrm{med},\;q_{0.1},\;q_{0.9},\;\mathrm{rms},\;E,\right.\\ \; & \;{\left.\mu_{f},\;\sigma_{f},\;M_{\max},\;\mathrm{dom}\right]}^{⊤}\in R^{13}, \end{array}$$

where the frequency-domain terms are computed from the single-sided magnitude spectrum |RFFT(x)|. Concatenating the descriptors from all IMU channels gives the window-level feature vector

(5) $$\begin{array}{cc} w_{k} & =\mathrm{Concat}\left(\psi \left(x^{\left(k,0\right)}\right),\ldots ,\psi \left(x^{\left(k,D_{0}-1\right)}\right)\right)\in R^{d},\;d=13D_{0}. \end{array}$$

After standardization and PCA compression, each window is represented by $z_{k}\in R^{m}$. A history tensor $X_{k}\in R^{H\times m}$ is then constructed from the most recent *H* past window embeddings and used as the causal input to the temporal prediction model.

#### 3.2.2. Class Imbalance Handling

The *other* label constitutes the majority of window samples. We therefore perform random undersampling: within each file, we retain all non-*other* windows and randomly keep an equal number of *other* windows (when non-*other* windows exist); files containing no non-*other* windows are excluded from balancing. After balancing, we randomly split the remaining windows into train/validation/test sets with a 70/10/20 ratio (fixed seed for reproducibility).

#### 3.2.3. PCA-Based Dimensionality Reduction with Feature Standardization

To alleviate scale mismatch and feature redundancy, we apply feature standardization followed by PCA. Specifically, we fit a standard scaler on the training split only and transform the validation/test splits using the same parameters to avoid information leakage. PCA is then fitted on the standardized training features, and all splits are projected into the PCA space. We set the PCA dimension by retaining a target explained-variance ratio (e.g., 95%) or by selecting the best-performing dimension on the validation set. In practice, this compresses correlated statistics (e.g., energy-related measures) into a smaller set of orthogonal components and improves downstream training stability.

#### 3.2.4. Causal Temporal Prediction Architecture

The temporal model maps a history of past IMU window embeddings to the action label distribution of one or more future windows. For a window index *k*, the causal input is the history tensor

(6) $$X_{k}=\left[z_{k-H+1},\ldots ,z_{k}\right]\in R^{H\times m},$$

where H=history_len is the number of historical windows and m=enc_in is the PCA-reduced feature dimension. The corresponding supervision is defined over a prediction horizon,

(7) $$y_{k}^{\mathrm{tar}}=\left[y_{k+f_{1}},\ldots ,y_{k+f_{K}}\right]\in {\left\{0,\ldots ,C-1\right\}}^{K},$$

where $f_{s}\in \left[\mathrm{future}\_\mathrm{start},\mathrm{future}\_\mathrm{end}\right]$ and K=future_end−future_start+1. This formulation separates the observed history from the future target labels and therefore preserves the past-only constraint required for streaming deployment.

For mini-batch training, the input tensor is $X\in R^{B\times H\times m}$ and the target tensor is $Y^{\mathrm{tar}}\in {\left\{0,\ldots ,C-1\right\}}^{B\times K}$. The network produces horizon-wise logits

(8) $$O=F_{\theta }\left(X\right)\in R^{B\times K\times C},\;{\hat{y}}_{i,s}=\arg\max_{c}O_{i,s,c}.$$

The first stage projects each time-step feature vector $x_{\tau }\in R^{m}$ into the model dimension d=d_model,

(9) $$h_{\tau }=\mathrm{Dropout}\left(W_{in}x_{\tau }+b_{in}\right)\in R^{d},$$

which yields a sequence representation $H\in R^{B\times H\times d}$. This projected sequence is encoded by L=depth stacked bidirectional LSTM layers with hidden size h=d_hidden, allowing the model to summarize short-term stroke variations together with broader temporal context within the available history.

The BiLSTM output is then projected back to the model dimension and refined by multi-head self-attention (MHSA). After LayerNorm and a residual connection, the refined sequence is

(10) $$U=\mathrm{LN}\left(Z+\mathrm{MHSA}\left(Z\right)\right),\;Z\in R^{B\times H\times d}.$$

A fixed-length causal representation is obtained by last-step pooling,

(11) $$u=U_{:,H-1,:}\in R^{B\times d},$$

so the classifier uses the representation at the most recent available historical window. A feed-forward MLP maps this representation to KC logits and reshapes them into horizon-specific class scores,

(12) $$r=\mathrm{MLP}\left(u\right)\in R^{B\times (KC)},\;O=\mathrm{Reshape}\left(r\right)\in R^{B\times K\times C}.$$

This non-autoregressive prediction head estimates all horizon steps in a single forward pass, which reduces inference overhead and supports real-time use.

During training, the horizon dimension is flattened so that each horizon step contributes one classification term:

(13) $$L=\frac{1}{BK}\sum_{i=1}^{B}\sum_{j=1}^{K}L_{\mathrm{LS}}\left(O_{i,j,:},y_{i,j}\right),$$

where $L_{\mathrm{LS}}$ denotes label-smoothed cross-entropy with ε=label_smoothing. Together with PCA compression and fixed-size sliding windows, this architecture is designed to balance temporal modeling capacity with the throughput requirements of real-time badminton analytics.

**Figure 2:**
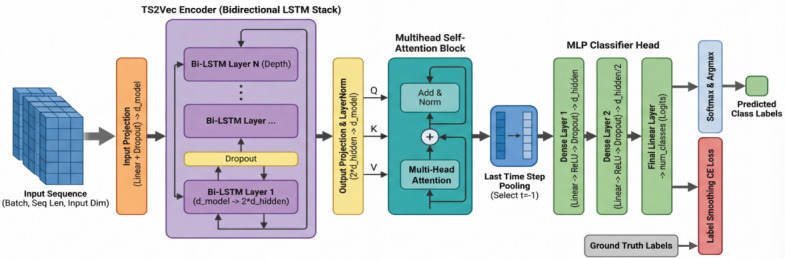
Overall architecture of the real-time badminton model. The past-only multivariate input sequence is first projected into a latent feature space, which is followed by stacked bidirectional LSTM layers for temporal encoding. Multi-head self-attention is then applied to capture global temporal dependencies, and the refined representation is finally mapped to class logits through a feed-forward classification head. Here, $d_{\mathrm{model}}$ and $d_{\mathrm{hidden}}$ denote feature dimensions, and the last-time-step pooling selects the final encoded state.

The architecture of the proposed model is illustrated in Figure 2.

### 3.3. Self-Supervised Learning Approach

To generate reliable labels for the IMU sensor data of badminton players, we adopt a self-supervised learning-based annotation method, which is derived from the LIMU-BERT framework proposed by Xu et al. [21]. Unlike manual annotation that relies on experience, this self-supervised approach (originally designed for IMU sensing tasks) enables automatic label generation by leveraging intrinsic time-series characteristics of wearable sensor data, significantly reducing human labor costs while ensuring label consistency—aligning with the core advantage of LIMU-BERT in mining unlabeled sensor data.

## 4. Results and Analysis

A series of experiments were conducted to verify the proposed method. This section presents the core results obtained in the experiment with a focus on analyzing the performance of real-time prediction models in highly dynamic IMU data. The subsequent analysis provided a solid foundation for verifying the effectiveness and superiority of the proposed method.

### 4.1. Window-Level Offline Evaluation

We evaluate the proposed model on the window-level next-window prediction task (prediction horizon = 1 step). Since the dataset is class-imbalanced (e.g., the *other* class has substantially larger support), we report Macro-F1 and balanced accuracy in addition to overall accuracy. Table 1 summarizes the overall performance, and Table 2 provides per-class precision/recall/F1, which enables a fine-grained inspection of class-wise strengths and failure modes.

### 4.2. Performance of the Optimized LSTM Model

**Figure 3:**
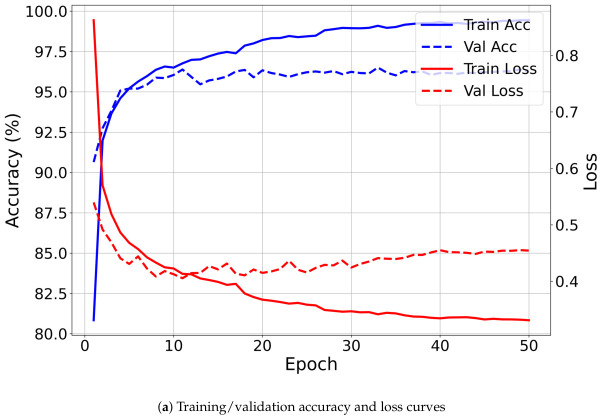

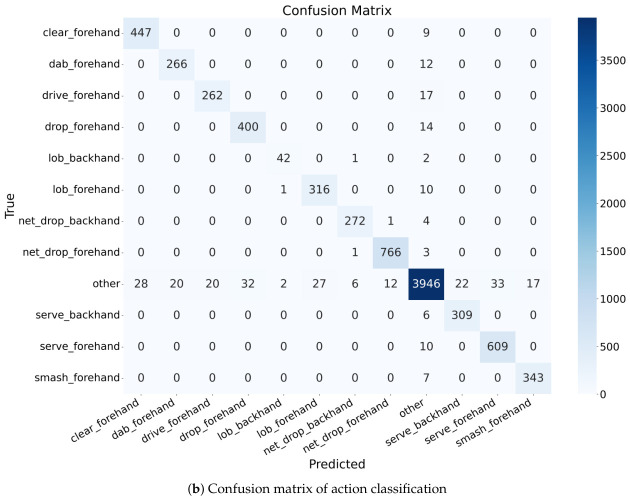
Performance of the optimized LSTM-PCA model (w=10, hop=4, H=49, d=52). (**a**) Training/validation accuracy and loss curves. (**b**) Confusion matrix on the test set.

To validate the effectiveness of the combined hyperparameter optimization strategy, we integrate the optimal temporal configuration (w=10, hop=4, H=49) and the recommended PCA dimensionality (d=52) to construct the final LSTM model. Figure 3 presents key performance visualizations of this integrated model, including training/validation accuracy, class distribution balance, confusion matrices, and loss dynamics.

To avoid repeating the aggregate metrics already reported in *Window-Level Offline Evaluation*, we focus here on optimization dynamics and class-wise error patterns. In Figure 3a, the training/validation curves remain close throughout optimization, indicating stable convergence and no obvious overfitting under the selected configuration.

Figure 3b further shows that most confusion is concentrated among semantically similar stroke classes, while the dominant “other” class remains well separated rather than being over-predicted. The main residual weakness is the rare *lob_backhand* class (very limited support), which is consistent with the long-tail distribution and suggests that future gains will primarily come from targeted data balancing or class-aware augmentation rather than further global hyperparameter tuning.

To further contextualize the IMU-only setting, we discuss its relation to prior wearable-sensing-based badminton recognition work without treating it as a directly comparable benchmark.

### 4.3. End-to-End Real-Time Inference Benchmark

A system is considered real-time feasible in our setting if throughput is at least 10 windows/s, since the deployment hop is 100 ms. Our end-to-end pipeline achieves 58.20 windows/s, i.e., 5.82× the real-time requirement. To verify this under realistic processing overhead, we run an end-to-end stream-replay benchmark. Raw IMU CSV streams are replayed in chronological order, and the pipeline performs sliding-window inference per hop, including window-level feature extraction, standardization, PCA projection, history buffering, and a single model forward pass. After warming up for $N_{w}$ windows, we benchmark $N_{e}$ consecutive windows and report throughput and latency percentiles.

**Metrics.** Let $f_{s}$ be the sampling rate (Hz) and let $h_{\mathrm{out}}$ denote the output hop size (samples). The system produces one prediction every $\Delta t=h_{\mathrm{out}}/f_{s}$ seconds; thus, the minimum required throughput is

(14) $${\mathrm{TP}}_{\min}=\frac{1}{\Delta t}=\frac{f_{s}}{h_{\mathrm{out}}}\;\left(\mathrm{windows}/s\right).$$

Given the measured throughput TP (windows/s), we define the real-time factor (RTF) with respect to the output hop as

(15) $${\mathrm{RTF}}_{\mathrm{hop}}=\frac{\mathrm{TP}}{{\mathrm{TP}}_{\min}}=\mathrm{TP}\cdot \frac{h_{\mathrm{out}}}{f_{s}}.$$

A system is considered real-time feasible if ${\mathrm{RTF}}_{\mathrm{hop}}>1$.

**Results and analysis.** With $f_{s}=100$ Hz and $h_{\mathrm{out}}=10$ (i.e., Δt=0.1 s, ${\mathrm{TP}}_{\min}=10$ windows/s), our end-to-end pipeline achieves TP=58.20 windows/s, corresponding to ${\mathrm{RTF}}_{\mathrm{hop}}=5.82$, which satisfies the real-time requirement with a clear safety margin. In addition, model-only inference (batch size 1) reaches 185.17 windows/s with median latency p50=5.08 ms, indicating that the remaining end-to-end overhead mainly stems from preprocessing (feature computation, normalization, and PCA) rather than the network forward pass. This margin suggests the system can tolerate moderate deployment overhead (I/O, scheduling, logging) while still meeting the hop-based real-time constraint.

### 4.4. Calibration and Selective Prediction

Beyond accuracy, deployment quality depends on whether confidence scores are well calibrated and useful for selective prediction. We therefore evaluate reliability with the Expected Calibration Error (ECE) and assess confidence ranking quality with precision–recall analysis. Using *B* confidence bins, ECE is defined as

(16) $$\mathrm{ECE}=\sum_{b=1}^{B}\frac{|I_{b}|}{N}\left|\mathrm{acc}\left(I_{b}\right)-\mathrm{conf}\left(I_{b}\right)\right|,$$

where $I_{b}$ is the sample set in bin *b*, *N* is the number of test windows, $\mathrm{acc}(I_{b})$ is the empirical accuracy, and $\mathrm{conf}(I_{b})$ is the mean predicted confidence; we set B=15 in this paper. A lower ECE indicates better alignment between predicted probabilities and true correctness frequencies.

**Figure 4:**
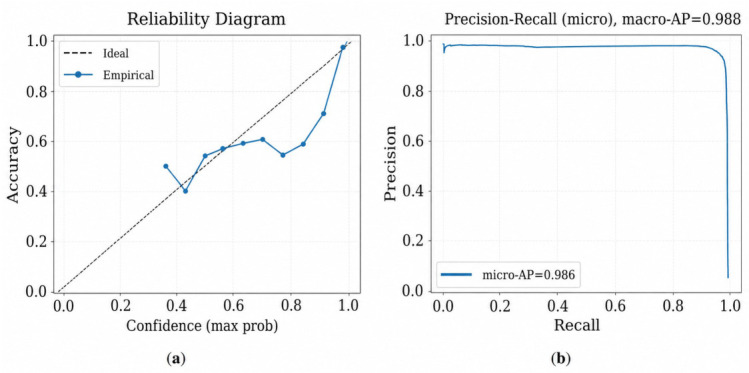
Calibration and confidence-ranking diagnostics on the test set: (**a**) reliability diagram with the dashed ideal-calibration line; (**b**) micro precision–recall curve showing strong confidence ranking quality.

Figure 4a shows that the reliability curve closely follows the diagonal, indicating good calibration. For the reliability diagram (Figure 4a), the *x*-axis is the binned mean confidence ${\bar{c}}_{b}\in \left[0,1\right]$, and the *y*-axis is the corresponding empirical accuracy $\mathrm{acc}\left(I_{b}\right)\in \left[0,1\right]$ for bin $I_{b}$. Figure 4b further shows strong precision–recall behavior, suggesting that confidence can effectively separate likely-correct from likely-incorrect predictions. For the PR curve (Figure 4b), the *x*-axis is recall $R=\frac{TP}{TP+FN}$ and the *y*-axis is precision $P=\frac{TP}{TP+FP}$. Moreover, the micro-AP is 0.986 and macro-AP is 0.988, indicating strong confidence ranking quality. Precision decreases when recall approaches 1 because lowering the decision threshold introduces more low-confidence false positives. Together, these results support confidence-based deployment strategies (e.g., thresholding or abstention) in streaming scenarios.

### 4.5. Ablation Studies

**Figure 5:**
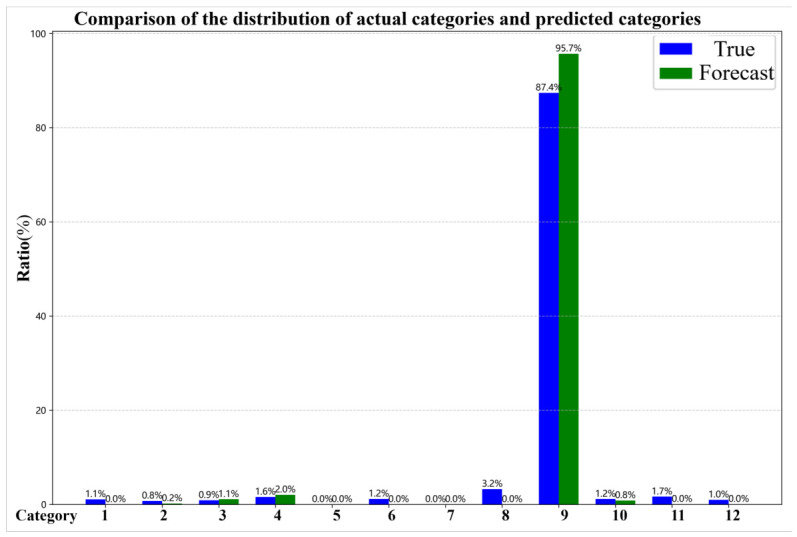
Class distribution comparison before and after downsampling.

**Downsampling under severe class imbalance.** The raw window-level labels are highly imbalanced where the *other* class dominates. Without handling this imbalance, the classifier tends to collapse to a trivial majority-class predictor, yielding misleadingly high accuracy while failing on minority actions. Therefore, we apply downsampling to mitigate the dominance of the majority class and stabilize optimization. Figure 5 provides a class-distribution comparison before and after downsampling.

**Effect of PCA.** We further evaluate whether PCA-based dimensionality reduction benefits horizon prediction. Table 3 reports the mean accuracy (averaged over all steps within the prediction horizon) under four prediction horizons. PCA consistently improves performance for short-to-medium horizons (10–40), indicating that denoising and compact representations help the encoder learn more robust temporal features.

**Effect of MHSA.** We ablate the multi-head self-attention (MHSA) module by removing it from the encoder while keeping all other settings unchanged. As shown in Table 4, MHSA provides consistent gains across different horizons, especially at longer horizons, which suggests that attention helps highlight informative temporal segments within the historical window for more reliable prediction across horizons.

**Figure 6:**
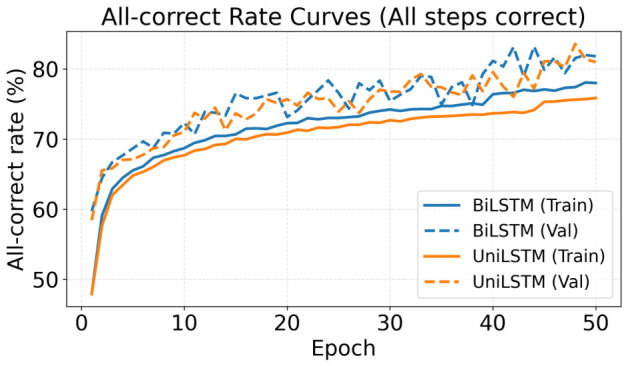
BiLSTM vs. UniLSTM ablation on horizon prediction (horizon length 20). The metric is the all-correct rate over epochs for train/validation splits.

**Figure 7:**
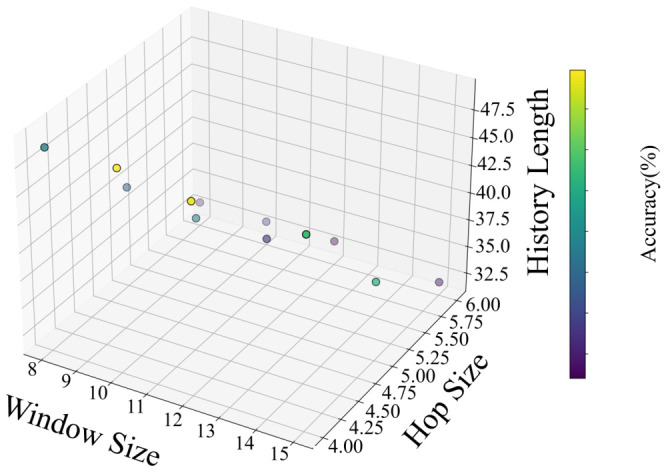
Grid search results for LSTM model accuracy: optimal parameters are w=10, hop=4, hist=49.

**Figure 8:**
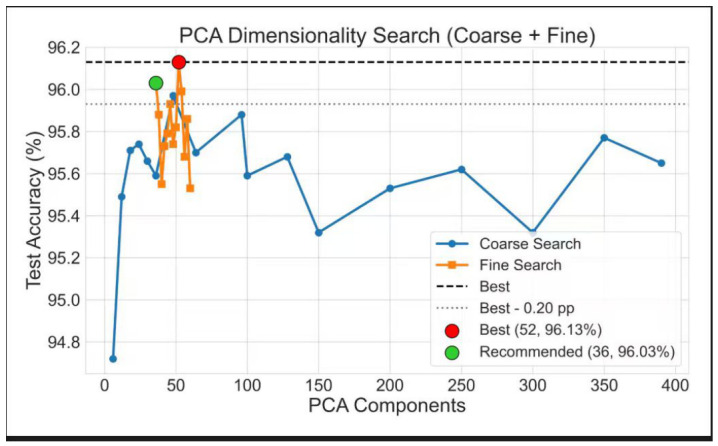
PCA dimensionality analysis (combined): coarse and fine accuracy curves, best-per-dimension accuracy envelope, and accuracy gap to the best.

**BiLSTM vs. UniLSTM.** Finally, we compare a bidirectional LSTM encoder with a unidirectional LSTM encoder under the same training protocol. Note that the bidirectionality is applied *only within the observed input window* and does not access any future observations from the prediction horizon; hence, it does not introduce information leakage. Figure 6 visualizes the all-correct rate (the percentage of samples for which all steps within the prediction horizon are correctly predicted) over training. BiLSTM achieves a higher all-correct rate throughout training, suggesting that modeling both earlier and later temporal dependencies *inside the input window* yields a more informative representation for prediction across the horizon. Additional ablation details are provided in Figure 7 for key-parameter sensitivity and in Figure 8 for PCA dimensionality analysis.

## 5. Conclusions and Future Work

### 5.1. Conclusions

This paper studied real-time badminton *next-window* action prediction from wearable IMU streams under a strict past-only protocol. We proposed a practical pipeline that combines sliding-window feature extraction, standardization and PCA compression, and a lightweight temporal model (BiLSTM with multi-head self-attention) to capture both short- and long-range motion context. Experiments on a multi-player, multi-sensor badminton dataset demonstrate strong recognition performance under severe class imbalance, while the end-to-end stream-replay benchmark confirms real-time feasibility (58.20 windows/s including preprocessing). Overall, the results indicate that compact IMU representations together with causal temporal modeling can enable accurate and deployable online action prediction for badminton analytics.

### 5.2. Future Work

Future extensions will focus on moving from action-level prediction toward richer real-time badminton intelligence. One direction is to incorporate UWB-based positional information so that body-motion cues from IMUs can be combined with on-court trajectories for strategy-level forecasting, including player movement intent, court coverage, and tactical patterns. This extension also requires multimodal fusion architectures that can align asynchronous IMU and UWB streams while remaining robust to realistic noise, occlusion, and sensor dropouts. Beyond sensing and prediction, large language models (LLMs) may serve as higher-level reasoning and generation modules: real-time predicted actions and positions could be translated into coherent play-by-play descriptions, tactical summaries, and short-horizon forecasts, making the system output more interpretable for players, coaches, and spectators.

## 6. Additional Analyses

This section provides additional analyses, including key-parameter sensitivity, PCA dimensionality effects, calibration and selective prediction, downsampled streaming/event-level evaluation, self-supervised labeling assessment, and deployment-oriented confidence analysis.

### 6.1. Impact of Key Parameters on Recognition Performance

To identify the optimal hyperparameter combination for the LSTM model, we conducted a grid search over three key parameters: window size (w), model hop size (hop), and history length (hist). The grid search results are visualized in Figure 7, which quantifies the model accuracy across different parameter combinations. Here, hop denotes the model-window stride used for feature sequence construction, which is distinct from the 100 ms deployment hop used in the real-time benchmark.

As indicated by the peak accuracy in Figure 7, the optimal parameter combination is determined as w=10 (window size), hop=4 (model hop size), and hist=49 (history length). A small window size (w=10) captures fine-grained dynamic features of badminton motions (e.g., rapid stroke transitions) without over-smoothing short-term IMU signal fluctuations. A small model hop size (hop=4) ensures dense sampling of the time series, preserving temporal continuity and reducing information loss between adjacent windows. A large history length (hist=49) enables the LSTM model to leverage long-term contextual dependencies of motion sequences, which is critical for distinguishing similar badminton shots (e.g., forehand clear vs. smash) that share short-term IMU patterns but differ in long-term motion context.

### 6.2. Impact of PCA Dimensionality on Recognition Performance

We investigated the impact of PCA dimensionality on recognition performance while keeping the temporal configuration fixed (w=10, hop=4, H=49) and using the same split protocol. We first conducted a coarse scan over a broad range of PCA components, which is followed by a fine-grained scan around the promising region. Across all evaluated settings, the best observed test accuracy was 96.13% at d=52 PCA components. To balance accuracy and model efficiency, we also report the smallest dimensionality whose accuracy is within 0.20 percentage points the best result; this yields a recommended setting of d=36 with 96.03% accuracy. Overall, PCA enables substantial dimensionality reduction with a negligible drop in accuracy, indicating that the handcrafted time/frequency features contain considerable redundancy and can be compactly represented without sacrificing recognition performance. The combined figure consolidates the coarse and fine accuracy curves, the best-per-dimension accuracy envelope, and the accuracy gap to the best, enabling a compact view of the accuracy–efficiency trade-off and the diminishing returns beyond the recommended dimensionality.

### 6.3. Calibration and Selective-Prediction Protocol

Calibration analysis evaluates whether the model confidence reflects the empirical probability of correct prediction, which is important when the system is used for real-time coaching or tactical feedback. For each test window, the model produces logits $z\in R^{C}$ and class probabilities p=softmax(z). The predicted label is $\hat{y}=\arg\max_{c}p_{c}$, and the associated confidence is $\max_{c}p_{c}$. These quantities support Expected Calibration Error (ECE), coverage–risk analysis, and confidence-thresholded selective prediction where low-confidence windows can be abstained from rather than forced into potentially unreliable action decisions. Table 5 reports the calibration result on the test set.

### 6.4. Streaming/Event-Level Evaluation (Downsampled Setting)

To better reflect event detection performance under controlled class imbalance, we additionally evaluate streaming predictions in a downsampled setting. Following our training-time strategy, we downsample background (*other*) windows to match the number of non-*other* windows (1:1) before constructing history sequences and running inference. We then merge consecutive non-*other* windows into predicted action segments and match them to ground-truth segments using temporal IoU with threshold θ=0.5. We report event-level precision/recall/F1 and detection delay measured by time-to-detect (median and 90th percentile), where delays are computed based on N=2 consecutive correct predictions at the native resolution of this event-level protocol. Table 6 summarizes the event-level performance in the downsampled streaming setting.

We further report the sensitivity to the temporal IoU threshold by sweeping θ∈{0.5,0.6,0.7,0.8}, as summarized in Table 7.

This downsampled setting reduces the dominance of the *other* class and therefore provides an upper-bound estimate of event detection performance under a balanced background.

### 6.5. Performance Evaluation of Self-Supervised Labeling

**Figure 9:**
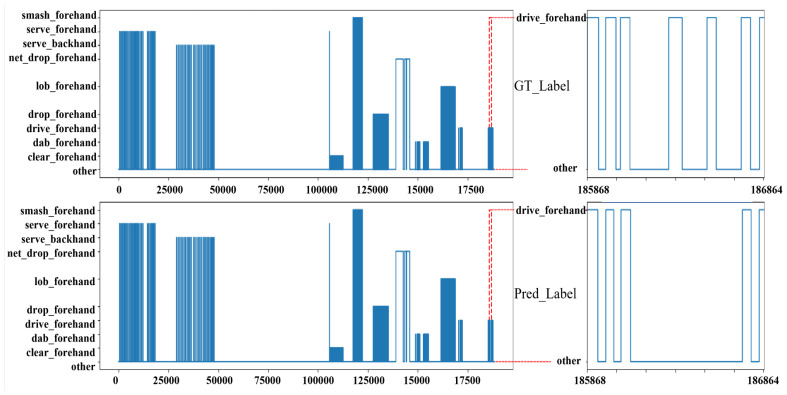
Self-supervised labeling for Player 3: GT vs. predicted labels with a side zoom of the largest discrepancy segment ((**top**) GT, (**bottom**) Pred). Accuracy: 0.9913.

In this experiment, the comparison between ground truth (GT) labels and self-supervised predicted (pred) labels for three players is visualized in Figure 9. Each player’s results include a side-by-side comparison of GT labels (left column) and model-generated labels (right column), enabling both a qualitative and quantitative assessment of the self-supervised labeling effectiveness.

Quantitative evaluation reveals that the self-supervised model achieves high labeling accuracy across all participants: Player 1 reaches 0.9888, Player 2 0.9753, and Player 3 the highest at 0.9913. Qualitatively, the predicted labels in Figure 9 closely align with the GT labels, accurately capturing the temporal boundaries of badminton actions (e.g., serves, smashes, and drops) without obvious misclassifications. This validates the model’s ability to generate reliable labels without manual annotation, significantly reducing labor costs.

Notably, window size configuration is critical for balancing labeling quality and temporal responsiveness. An excessively small window leads to label “glitches” (spurious action transitions) due to heightened sensitivity to short-term IMU signal noise—this is because small windows fail to average out random fluctuations in sensor data. Conversely, an overly large window introduces substantial labeling delay, as it requires accumulating more temporal data before generating a label, which cannot keep pace with the rapid dynamics of badminton motions (e.g., quick stroke reversals or sudden direction changes). The window size adopted in this paper optimizes this trade-off: as evidenced by the smooth label sequences and high accuracy in Figure 9, it effectively minimizes noise-induced glitches while maintaining sufficient temporal responsiveness to match the fast-changing characteristics of IMU data.

### 6.6. Deployment-Oriented Additional Analyses

To complement the main streaming and calibration results, we provide two additional analyses that are directly relevant to real-time deployment. The first examines confidence-based selective prediction, where the system may abstain from low-confidence windows rather than forcing unreliable decisions. The second evaluates the sensitivity of event-level performance to the temporal IoU threshold used for segment matching. Together, these analyses clarify how the proposed system behaves under practical confidence filtering and event-detection criteria.

**Figure 10:**
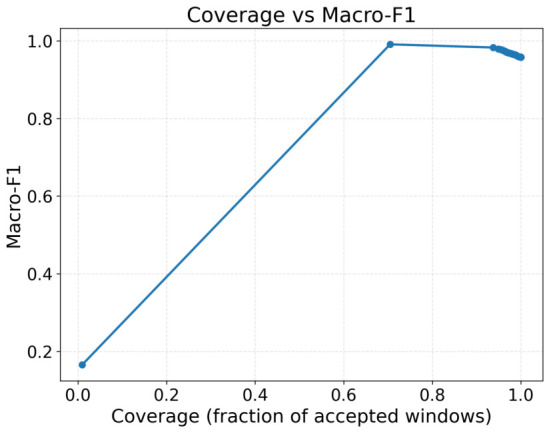
Coverage–risk curve under confidence thresholding. As the confidence threshold increases, coverage decreases while Macro-F1 improves, indicating that abstention effectively filters low-confidence errors.

Figure 10 shows the trade-off between prediction coverage and risk under confidence thresholding. This result supports selective deployment modes in which uncertain predictions can be withheld or flagged for downstream review, thereby improving the reliability of the displayed feedback.

Table 7 reports the event-level sensitivity to the temporal IoU threshold. As expected, stricter matching thresholds reduce precision, recall, and F1 because predicted segments must align more tightly with ground-truth action intervals. The gradual performance decrease indicates that the predicted event boundaries remain reasonably stable across a range of temporal matching criteria.

## Figures and Tables

**Table 1:** Window-level test performance (prediction horizon = 1 step).

Metric	Value
Accuracy	0.9636
Macro-F1	0.9582
Weighted-F1	0.9636
Balanced Accuracy	0.9654

**Table 2:** Per-class window-level metrics on the test set (prediction horizon = 1 step).

Class	Precision	Recall	F1	Support
clear_forehand	0.9314	0.9825	0.9562	456
dab_forehand	0.9397	0.9532	0.9464	278
drive_forehand	0.9362	0.9462	0.9412	279
drop_forehand	0.9585	0.9493	0.9539	414
lob_backhand	0.9149	0.9556	0.9348	45
lob_forehand	0.9181	0.9602	0.9387	327
net_drop_backhand	0.9926	0.9675	0.9799	277
net_drop_forehand	0.9846	0.9974	0.9910	770
serve_backhand	0.9464	0.9524	0.9494	315
serve_forehand	0.9472	0.9855	0.9660	619
smash_forehand	0.9744	0.9800	0.9772	350
other	0.9728	0.9549	0.9638	4165

**Table 3:** Ablation study results of PCA (mean accuracy).

Model	Prediction Horizon			
	10	20	40	80
No PCA	0.899	0.799	0.657	0.519
With PCA	0.915	0.825	0.686	0.519

**Table 4:** Ablation study results of MHSA (mean accuracy).

Model	Prediction Horizon			
	10	20	40	80
No MHSA	0.903	0.782	0.656	0.515
With MHSA	0.915	0.825	0.686	0.519

**Table 5:** Calibration on the test set.

Metric	Value
ECE (bins = *B*)	0.0267

**Table 6:** Event-level performance in the downsampled streaming setting (IoU θ=0.5).

Setting	Prec.	Rec.	F1	Med. (ms)	P90 (ms)
Downsampled (1:1)	0.959	0.889	0.923	0	40

**Table 7:** Event-level results under different temporal IoU thresholds θ in the downsampled streaming setting.

IoU θ	Precision	Recall	F1
0.5	0.959	0.889	0.923
0.6	0.942	0.873	0.906
0.7	0.921	0.854	0.887
0.8	0.865	0.802	0.832

## Data Availability

The data used in this study are available from the corresponding author upon reasonable request.
